# Cytomegalovirus infection in pediatric rheumatic diseases: a review

**DOI:** 10.1186/1546-0096-8-17

**Published:** 2010-05-20

**Authors:** Eli M Eisenstein, Dana G Wolf

**Affiliations:** 1 Department of Pediatrics, Hadassah-Hebrew University Medical Center, POB 24035, Mount Scopus, Jerusalem 91240, Israel; 2 Department of Clinical Microbiology & Infectious Diseases, Hadassah-Hebrew University Medical Center, Ein Kerem, Jerusalem, Israel

## Abstract

Human cytomegalovirus (HCMV) is familiar to pediatric rheumatologists mainly as a cause of opportunistic disease in pharmacologically immune suppressed patients. However, HCMV also has a variety of immuno-modulatory effects, through which it may influence the course of rheumatic conditions. In this article we discuss the interplay between HCMV and the immune system, and review the clinical manifestations, diagnosis, and treatment of HCMV infection in children with rheumatic disease.

## Introduction

Human cytomegalovirus (HCMV) is a ubiquitous beta-herpesvirus that causes symptoms primarily in immune-compromised individuals. Fully assembled virus is comprised of a DNA-containing capsid surrounded by a tegument layer and lipid envelope. The viral genome contains over 200 open reading frames [1].

HCMV is well-known to pediatric rheumatologists as an opportunistic pathogen. However, this virus also possesses immuno-modulatory properties, and may be a co-factor in the pathogenesis of certain forms of rheumatic disease. In this review, we discuss clinical and immunologic aspects of HCMV infection relevant to the practice of pediatric rheumatology.

### Epidemiology of HCMV infection

Sero-epidemiologic data indicate that between 30-70% of children in the United States have been infected with HCMV by school age [2]. The prevalence of anti-HCMV IgG antibodies is higher in minority children and those in developing countries, and continues to increase throughout life [3]. Humans are the only known host for HCMV, and infection requires contact with infected secretions. Common routes of infection include horizontal passage among family members, sexual transmission, vertical intra-uterine transmission, and breastfeeding. Children may continue to secrete virus for months or even years following primary infection, making them an important reservoir of infectious virus. Infection may be acquired though sexual contact in adolescents and adults [4]. Vertical transmission from mother to fetus can occur throughout pregnancy. Primary infection during the first trimester confers the greatest risk for severe congenital disease [5]. Transfusion of infected blood products and transplantation of infected organs are important sources of HCMV infection in the nosocomial setting.

### HCMV latency and re-activation

HCMV exists in two forms: as an actively replicating virus, and in a latent form. Latency is defined as persistence of non-replicating viral DNA in cells, that has the potential to re-activate and form infectious virus under propitious conditions [6]. While replicating *in vivo*, HCMV is capable of producing lytic infection in numerous cell types, including epithelial and endothelial cells, fibroblasts, and dendritic cells. In contrast, latent HCMV appears to be restricted mainly to myeloid-derived monocyte and dendritic cell precursors [7], including CD34+ pluripotent cells [8], although it is possible that latent virus is also present in other tissues. The tissue-specificity of latent HCMV is clinically significant, in that filtration of blood products to remove nucleated cells reduces the likelihood of HCMV transmission to transfusion recipients. Viral re-activation requires cellular differentiation, which in turn is influenced by various cytokines [9]. The principal molecular determinant of viral activation from the latent state is expression of the immediate early (IE) gene, which is under the control of the major IE promoter/enhancer (MIEP). MIEP activity is regulated by several activating and inhibiting transcription factors that are expressed in myeloid cells [10].

The pro-inflammatory cytokine tumor necrosis factor-α (TNFα) enhances replication of HCMV in monocytes, and this effect is potentiated by interferon-γ [11]. Moreover, high serum concentrations of TNFα are associated with increased viral load in recipients of solid organ [12] or bone marrow transplants [13]. Thus, in general, a pro-inflammatory cytokine milieu appears to favor HCMV re-activation. Pediatric rheumatic diseases with a systemic component are also characterized by increased concentrations of pro-inflammatory cytokines in the peripheral blood, [14-16], suggesting that the likelihood of clinically significant HCMV infection may be increased in the setting of uncontrolled rheumatic disease.

### HCMV-Immune Interactions

Both innate and adaptive immune mechanisms are essential for control of HCMV. The importance of cellular immunity is apparent from the frequency and severity of infections observed in patients with congenital or acquired cellular immune defects. Indeed, the extent of HCMV replication can be used as a barometer to gauge the degree of cellular immune suppression [17]. Both CD4+ and CD8+ T lymphocytes are required for full anti-HCMV immunity [18]. However, as would be expected for an intra-cellular pathogen, CD8+ T cell mediated immunity against antigen presented by Class I MHC molecules appears to be critical for control of viral replication. During viral latency, up to 10% of all CD8+ T cells in the peripheral blood may recognize HCMV epitopes [19]. These cells are comprised of relatively few clones that have expanded greatly [20]. The pp65 tegument protein is the most promiscuous target of the anti-HCMV T cell response [21], while appearance of anti-IE-specific CD8 T cell clones has also been correlated with protection against HCMV infection in solid organ transplant recipients [22].

The importance of natural killer (NK) cells in anti-CMV immunity is inferred from experiments in mice infected by murine cytomegalovirus (MCMV). In these studies, animals deficient in NK cell function are susceptible to severe MCMV disease. Anti-MCMV activity appears to be confined to a specific NK cell subset [23]. Cases of severe HCMV disease in humans with impaired NK cell function have been reported [24,25]. Anti-HCMV hyper-immune globulin appears to have some efficacy for prevention of primary HCMV infection in solid organ transplant recipients, and there is evidence that intravenous hyperimmune anti-HCMV globulin may prevent vertical transmission of HCMV during pregnancy [26]. These findings point to a role for humoral immunity in control of HCMV.

HCMV possesses a variety of mechanisms which enable it to evade the immune response during active replication and possibly during latency. The best characterized of these mechanisms is the capacity to down-regulate MHC Class I molecule expression [27]. Interestingly, MHC-I down regulation per se does not prevent emergence of a robust anti-HCMV CD8+ T cell response [28]. Numerous other mechanisms for evasion of cell-mediated immunity have been described, including inhibition of MHC-II expression [29] and expression of a viral homologue of the naturally occurring human immunosuppressive cytokine interleukin-10 [30].

HCMV is capable of inhibiting NK cell activation by several mechanisms [31]. Some of the more interesting of these include a specific inhibitory interaction between the HCMV pp65 tegument protein and an NK cell activating receptor, NKp30 [32], and a recently described form of NK cell inhibition via viral micro-RNA [33].

### Clinical HCMV disease in children

Primary HCMV infection in immune-competent children may sometimes result in an acute, self-limited mononucleosis-like illness, but more commonly is asymptomatic and goes unrecognized [1]. Re-infection by an antigenically un-related strain may also occur, with the same clinical consequences [34].

HCMV infection in immune-suppressed patients [35], including those with severe combined immune deficiency, acquired immune deficiency syndrome [36], or recipients of bone marrow [37] or solid organ transplants [38] is usually caused by re-activation of latent virus, and may cause severe, life threatening disease. As noted above, during active replication, HCMV is capable of infecting numerous types of cells, resulting in involvement of multiple organ systems including the lung, eye, intestine, and central nervous system. However, signs and symptoms of HCMV infection may also be subtle or non-specific, and a high index of suspicion may be required for prompt diagnosis.

### CMV infection in patients with rheumatic disease

HCMV re-activation is well-recognized in both adult and pediatric patients with rheumatic disease. In systemic lupus erythematosis (SLE), HCMV disease occurs mainly in individuals receiving potent immune suppressive regimens [39]. However, infection has also been described in patients receiving little or no medication to control their disease [40], suggesting that underlying immune dysregulation may predispose lupus patients to HCMV re-activation. Protean clinical sequelae, including retinitis [41], pneumonitis [42], hepatitis [43], esophagitis [40], enteritis [44], pancreatitis [45], and hematologic abnormalities [46] have been described, making it sometimes difficult to distinguish infection from a lupus flare on clinical grounds. Moreover, HCMV infection may trigger a lupus exacerbation [47,48], emphasizing the possibility that more than one disease process may be present in a given patient. HCMV infection in lupus is a potential trigger for macrophage activation syndrome (MAS, discussed further below) and may be associated with high rate of mortality [49], underscoring the need to consider HCMV along with other viral infections as a potential cause of unexplained clinical deterioration of a lupus patient.

Opportunistic HCMV disease has also been described in other rheumatic diseases that include a significant component of systemic illness. In recent multi-center survey which included adult and pediatric patients hospitalized for rheumatic diseases in Japan [50], HCMV infection was identified on the basis of a positive pp65 antigenemia test or histopathology in 151/7377 patients. Among those who tested positive for HCMV antigenemia, 117 were symptomatic. The most common rheumatic diagnoses were SLE, dermatomyositis, mixed connective tissue disease, rheumatoid arthritis, and various forms of systemic vasculitis. Many were receiving combination immunosuppressive treatment, including glucocorticoids and cytotoxic drugs. Significantly, however, 27 patients developed HCMV infection while receiving oral prednisone without other immunosuppressive drugs. High-dose pulse methylprednisolone treatment was associated with particularly high risk for HCMV infection.

Few data concerning the risk for HCMV infection conferred by individual immunosuppressive drugs other than glucocorticoids are available. However, re-activation of another herpes virus, varicella-zoster virus, during treatment for rheumatic diseases has been examined. In one study, the hazard ratio for zoster in adult patients receiving cyclophosphamide was 4.2, azathioprine 2.0, prednisone 1.5, leflunamide 1.4, and methotrexate 1.0 [51]. Fatal HCMV infection has been reported in a patient with juvenile idiopathic arthritis treated with azathioprine [52]; it is unknown whether this child was deficient of an enzyme critical for azathioprine metabolism, thiopurine methyltransferase. Leflunamide has anti-HCMV activity *in vitro *[53], but it is unclear whether this property outweighs its immune suppressive effects. Reports concerning whether infliximab treatment confers increased susceptibility to HCMV infection have yielded conflicting findings [54,55], but it appears that patients receiving TNFα antagonists may be at mildly increased risk for HCMV re-activation [56]. Information concerning the risk for HCMV disease posed by other biologic agents is limited. No cases of HCMV disease in patients receiving rituximab for rheumatic disease have been reported to date. However, rituximab has been associated with severe or fatal HCMV infections in lymphoma patients, and it has been suggested that oncologic patients should be monitored for HCMV during rituximab therapy [57,58].

### HCMV as a possible cause of arthritis

At present, there is little evidence to support a role for HCMV in the pathogenesis of arthritis. Low amounts of HCMV DNA have been detected in the peripheral blood [59] and joints [60] of adults with rheumatoid arthritis. The etiologic significance of these findings is unclear. The literature describing HCMV infection in pediatric rheumatic diseases is even more limited than in adult patients. In one study using non-quantitative PCR techniques, the prevalence of HCMV DNA in leukocytes obtained from children with SLE and JIA was found to be similar to controls [61].

### Possible role of HCMV in SLE and other autoimmune diseases

A number of studies have examined the possibility that HCMV infection may be a co-factor in the pathogenesis of SLE. In most, serologic methods were used to detect anti-CMV IgM and IgG antibodies. As discussed below, these tests may be unreliable in patients receiving immunosuppressive drugs. Furthermore, it is difficult to know whether detection of antibodies reactive with viral epitopes in individuals with an autoimmune disease is a cause or result of immune dysregulation.

With these caveats in mind, at least five reports in have examined anti-CMV antibodies in the sera of adult SLE patients [62-66]. With one exception [62], these studies have found that the prevalence of anti-CMV IgG antibodies is similar in lupus patients to that in the control population. In contrast, in four reports, the prevalence of anti-CMV IgM antibodies was increased in lupus patients as compared to controls, and in three [64-66] the increase was statistically significant. Several possible mechanisms for these findings have been proposed, including antigenic mimicry, epitope spreading, immune activation by a putative viral superantigen, polyclonal B cell activation, and viral re-activation in the context of immune suppression [67]. Only one small study examining HCMV viral load in SLE patients has been reported. In this study, HCMV copy number was not increased in the peripheral blood of SLE patients as compared to healthy control donors [68].

Other investigators have examined the possibility that specific HCMV epitopes may induce auto-antibodies with a potential role in the pathogenesis of SLE or mixed connective tissue disease. These studies were prompted in part by pre-clinical vaccine studies which showed that immunization of mice with a candidate HCMV vaccine expressing the HCMV UL-55 (glycoprotein B) antigen elicited production of anti-U1 70kd protein antibodies [69]. These auto-antibodies were not detected in human subjects following immunization with either of two candidate vaccines expressing CMV glycoprotein B antigen [70]. Reports examining the prevalence of anti-U1 70kd antibodies in anti-CMV adult sero-positive SLE patients have yielded conflicting results [71,72]. One recent study found that among SLE patients who are sero-positive for anti-CMV antibodies, antibodies against certain auto-antigens including U1 70kd protein are particularly frequent in patients who were anti-CMV IgG positive but anti-CMV IgM negative [73]. The significance of this finding is uncertain. In summary, while there appears to be an association between certain anti-CMV antibodies and SLE, further study will be required to elucidate whether they have any patho-physiologic significance.

MCMV has been shown to induce sialadenitis reminiscent of Sjögren's syndrome in C57Bl/6-lpr/lpr mice [74]. The relevance of this model to human disease is uncertain. Two cases of anti-phospholipid syndrome with deep venous thrombosis associated with anti-CMV IgM and IgG antibodies have been reported [75,76].

### CMV as a possible trigger of macrophage activation syndrome

Macrophage activation syndrome (MAS) is a potentially fatal condition characterized clinically by fever, lymphadenopathy, central nervous system involvement, hepatitis that may progress to fulminant hepatic failure, coagulopathy, and hemophagocytosis in the bone marrow. Evidence of occult MAS may be detected in as many as half of patients with systemic onset juvenile idiopathic arthritis (SJIA). MAS has also been described in SLE and other pediatric rheumatic diseases [77,49].

MAS closely resembles another syndrome of immune dys-regulation and inflammation, termed hemophagocytic lymphohistiocytosis (HLH) that occurs in both familial and sporadic forms. Familial HLH occurs as a result of genetic defects in molecules essential for normal NK cell function [78]. For this reason, NK cell function has also been studied in MAS. These studies revealed that as in HLH, NK cell function is impaired in approximately 50% of children with MAS [79], and more recent gene expression data have shown that NK-cell related genes are down-regulated in children with SJIA complicated by MAS [80]. Taken together, these findings suggest that NK cell defects may have an important role in the pathogenesis of MAS.

Rarely, polymorphisms in genes essential to normal NK cell function can be demonstrated in children with MAS [81]. However, in the majority of cases of SJIA- associated MAS there is no known genetic explanation for NK cell dysfunction. However, one possible hypothesis that might explain these NK cell abnormalities is immunomodulatory effects of herpes viruses. In this regard, using molecular techniques, HCMV or Epstein-Barr virus DNA can be demonstrated in the peripheral blood of about half of these patients [82]. As noted above, herpes viruses are capable of attenuating NK cell function through a variety of specific molecular interactions. Moreover, herpesviruses, including HCMV, Epstein-Barr virus, and human herpesvirus-6 are well recognized triggers for HLH [83]. Taken together, these observations suggest that herpesviruses including HCMV may be a trigger for MAS, and they should be sought in children with rheumatic disease who present with this complication.

### Laboratory diagnosis of HCMV infection

Serologic tests for anti-HCMV specific antibodies are used to diagnose primary infection in immune-competent persons. While the presence of anti-HCMV IgM antibody may indicate primary or recurrent infection, the value of this test is limited by sub-optimal sensitivity, discordance of results between different commercial kits, and variable persistence of antibody following primary infection. Furthermore, results may be influenced by the presence of rheumatoid factor or heterophile antibodies caused by infection by Epstein Barr virus [84]. Finally, as indicated above, the presence of anti-HCMV IgG antibodies does not imply full protective immunity against HCMV infection, since both viral re-activation and re-infection may occur. Serologic testing is not considered reliable for diagnosis of infection in the setting of compromised immunity [85].

The advent of direct antigenic and molecular testing has revolutionized the diagnosis of HCMV infection in the setting of immune suppression. HCMV pp65 antigenemia or quantitative polymerase chain reaction (PCR) based tests can be used for direct detection of virus in peripheral blood lymphocytes with semi-quantitative or quantitative measurement of viremia, respectively. Viral load has proven useful for diagnosis of infection, prediction of disease, and monitoring response to therapy [86,87]. One should keep in mind that detection of small amounts of viral DNA in peripheral blood by PCR is not necessarily indicative of clinically significant HCMV infection, and correct interpretation of test results may require consultation with the virology laboratory or an infectious diseases specialist. Further confirmatory diagnosis of organ specific involvement can be made by histologic detection of characteristic inclusion bodies contained in cells from bronchoalveolar lavage fluid, or tissue biopsy specimens. Other techniques for identifying HCMV in tissue include immunohistochemistry, in situ hybridization, and electron microscopy [88]. Confirmation of tissue infection by viral culture may be of interest in retrospect, but culture is usually too slow to be useful for patient management.

Treatment of clinically significant HCMV infection in a child with systemic rheumatic disease consists of reducing the level of immune suppression to the minimum necessary to avoid acute immune-mediated complications of disease, and specific anti-viral therapy. Three drugs are currently approved for treatment of severe HCMV disease: ganciclovir, foscarnet, and cidofivir. Each target the same molecule, the viral DNA polymerase. In general, the use of anti-HCMV drugs in clinical practice is currently limited by dose-dependent toxicity. Intravenous gancyclovir in considered the first drug of choice for severe disease. Emergence of resistant strains has been an increasing concern in the transplant setting. Most ganciclovir resistance is due to mutations in the HCMV UL97 or DNA polymerase genes [89]. Testing for these mutations is available in reference laboratories in situations when treatment fails to reduce viral load. Other anti-viral drugs can be used for treatment of resistant strains, although cross-resistance may develop. Recently, oral valganciclovir has been increasingly used as an alternative to intravenous ganciclovir for prophylaxis and treatment of HCMV disease in specific settings [90]. The role of this drug in the management of HCMV infection in patients with rheumatic disease remains to be defined.

## Summary

HCMV disease may occur as a consequence of re-activation of latent virus in children receiving immunosuppressive drugs for systemic rheumatic disease. Patients with uncontrolled inflammation who are receiving immunosuppressive medications appear to be at particularly high risk. Since the clinical manifestations of HCMV disease are protean, antigenic or PCR-based tests should be used to detect this virus in a child with systemic rheumatic disease who fails to respond as expected to immunosuppressive treatment. In addition, infection by HCMV and other human herpesviruses should be sought in children with macrophage activation syndrome, as these viruses are both common and potential triggers for this complication.

## Abbreviations

HCMV: Human cytomegalovirus; HLH: Hemophagocytic lymphohistiocytosis; IE: human cytomegalovirus early intermediate gene; MAS: Macrophage activation syndrome; MCMV: murine cytomegalovirus; MIEP: major early intermediate promoter/enhancer; NK cell: Natural Killer cell; PCR: Polymerase chain reaction; SJIA: Systemic onset juvenile idiopathic arthritis; SLE: Systemic lupus erythematosis;

## Competing interests

The authors declare that they have no competing interests.

## Authors' contributions

EME conceived of and wrote most of the review. DGW contributed sections concerning HCMV biology and diagnosis and treatment of HCMV infection in immune-compromised patients.
